# Extra Virgin Olive Oil Phenols Dilate the Rat Mesenteric Artery by Activation of BK_Ca2+_ Channels in Smooth Muscle Cells

**DOI:** 10.3390/molecules25112601

**Published:** 2020-06-03

**Authors:** Rossana D’Agostino, Laura Barberio, MariaCarmela Gatto, Innocenzo Muzzalupo, Maurizio Mandalà

**Affiliations:** 1 Department of Biology, Ecology & Earth Sciences, University of Calabria, 87036 Rende (CS), Italy; roxannedag@gmail.com (R.D.); laura.barberio90@gmail.com (L.B.); mariacarmelagatto91@hotmail.it (M.G.); 2 Research Centre for Olive, Citrus and Tree Fruit—Council for Agricultural Research and Economics, 87036 Rende (CS), Italy; innocenzo.muzzalupo@crea.gov.it

**Keywords:** extra virgin olive oil phenols, mesenteric artery, vasodilation, smooth muscle cells, BK_Ca2+_ channels

## Abstract

Accumulating evidence has shown the beneficial health effects of extra virgin olive oil (EVOO) consumption in reducing blood pressure and preventing the risk of developing hypertension. Some studies associate the hypotensive activity of EVOO to a minor component—the phenols. This study was designed to investigate the effects of EVOO phenols on the rat resistance mesenteric artery (MA) and to find out the possible vascular pathways involved. The experiments were carried out using a pressurized myograph, which allowed the effects of phenols on isolated MA to be tested under different conditions: (a) with endothelium removed; (b) with inhibition of nitric oxide synthase by N_ω_-Nitro-l-arginine methyl ester hydrochloride (l-NAME, 10^−4^ M) + N_ω_-Nitro-l-arginine (l-NNA, 10^−4^ M); (c) with inhibition of cyclooxygenase by indomethacin (10^−5^ M); (d) with inhibition of guanylate cyclase by 1*H*-[1,2,4]Oxadiazolo[4,3-a]quinoxalin-1-one (ODQ,10^−5^ M) or adenylate cyclase by 9-(Tetrahydro-2′-furyl)adenine (SQ, 10^−5^ M); (e) with depolarization by high potassium chloride (40 mM); and (f) with inhibition of the large conductance Ca^2+^–potassium channels (BK_Ca2+_) with paxilline (10^−5^ M). EVOO phenols induce vasodilation of the endothelium, mediated by a direct effect on smooth muscle cells (SMC) by activation of BK_Ca2+_ channels, an action by which phenols can regulate the vascular tone of the resistance artery. Phenols can be regarded as bioactive molecules that may contribute to the antihypertensive effects of EVOO.

## 1. Introduction

Extra virgin olive oil (EVOO) is one of the main components of the Mediterranean diet, which has been long known to exert beneficial health effects. However, only over the last two decades have numerous epidemiological studies demonstrated that EVOO can prevent hypertension [1,2], one of the major risk factors for cardiovascular disease [3,4]. In particular, studies in humans have shown that EVOO decreases blood pressure, with an inverse relationship to EVOO consumption. This beneficial effect has been attributed to EVOO’s high levels of monounsaturated fatty acids (MUFA) and phenol components, which are absent among various cooking oils [5,6,7].

An important role of the EVOO phenols in the regulation of the blood pressure was shown by Ruız-Gutierrez et al. 1996 [8], who compared the effect of two similar MUFA-rich diets (olive oil and high-oleic sunflower oil) in hypertensive women. The authors reported that only the EVOO-rich diet induced a significant reduction of blood pressure, suggesting a role of the minor olive oil components (the phenols) in blood pressure regulation. This study is in agreement with other recent studies showing a decrease in systolic blood pressure after high-phenolic olive oil consumption in comparison with low-phenolic olive oil in both health and hypertensive people [9,10]. The potential effect of EVOO phenols on blood pressure is supported by their high bioavailability. Studies in humans and rats have shown absorption of EVOO phenols after ingestion in a dose-dependent manner, with around 98% of phenols present in plasma and urine [11,12,13].

The high bioavailability and the significant advantages of EVOO rich in phenols in preventing high blood pressure lead the authors to hypothesize that EVOO phenols could act on vessels to reduce peripheral vascular resistance and counteract increases in blood pressure. Our study showed for the first time the effect of the EVOO phenols on resistance mesenteric artery (MA) and clearly demonstrated that these compounds act by regulating the contraction state of the smooth muscle cell (SMC) component of the vascular wall. This study contributes to information on the action of EVOO phenols on the resistance artery, which may explain their antihypertensive properties and suggest a potential use of these compounds in the treatment of hypertension.

## 2. Results

**Figure 1:**
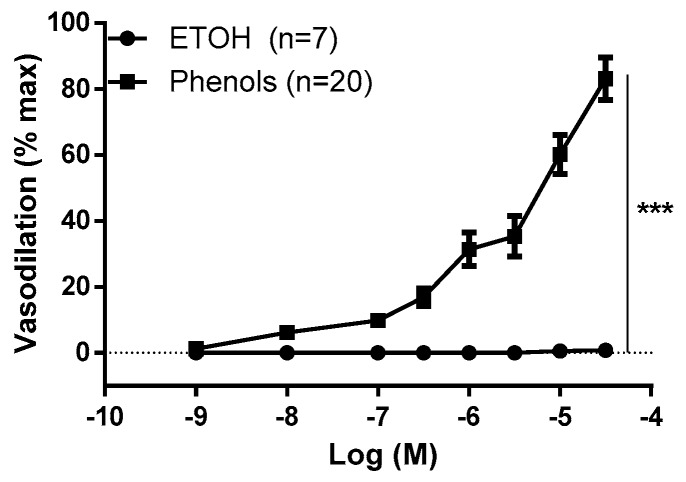
Extra virgin olive oil phenols dilate the resistance mesenteric artery (MA). Phenols from crude extra virgin olive oil (phenols) and the phenol vehicle, ethanol (ETOH), were tested on resistance mesenteric arteries isolated from rats. Data are reported as Mean ± SEM, n (experimental number). Statistical analysis was performed using two-way ANOVA, *** *p* < 0.001.

We tested EVOO phenols on the MA in the concentration range of 10^−9^–3 × 10^−5^ M, the effects of which are shown in Figure 1. EVOO phenols dilated the artery in a concentration-dependent manner; at 10^−8^ M the vasodilation was 6.2% ± 2.1%, while an intense effect of 83.1% ± 7.7% was observed at 3 × 10^−5^ M. Meanwhile, ethanol, which was used as the vehicle for the phenols, did not have any effect.

**Figure 2:**
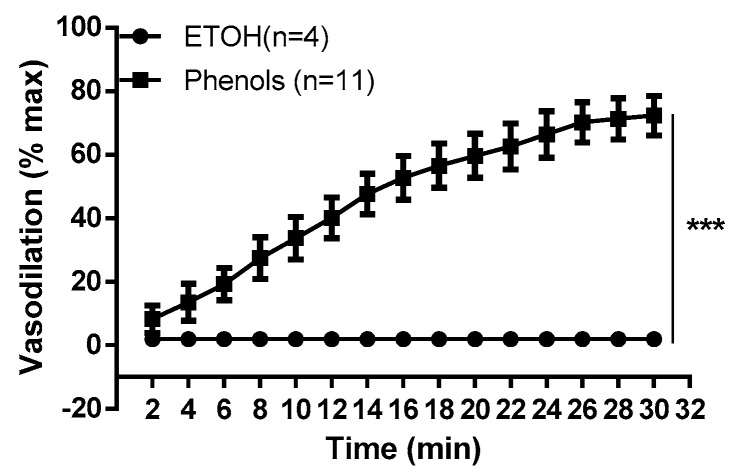
Time course of vasodilation induced by extra virgin olive oil phenols. The figure shows the time course for phenols (3 × 10^−5^ M) from crude extra virgin olive oil (phenols) and for the phenol vehicle, ethanol (1‰ vehicle), tested on phenylephrine-contracted resistance mesenteric arteries isolated from rats. Data are reported as the mean ± SEM, *n* (experimental number). Statistical analysis was performed using two-way ANOVA, *** *p* < 0.001.

The vasodilation of EVOO phenols was time-dependent and the maximum effect was reached within 30 min. For the same time period and conditions, no effect was observed for the ethanol (Figure 2).

**Figure 3:**
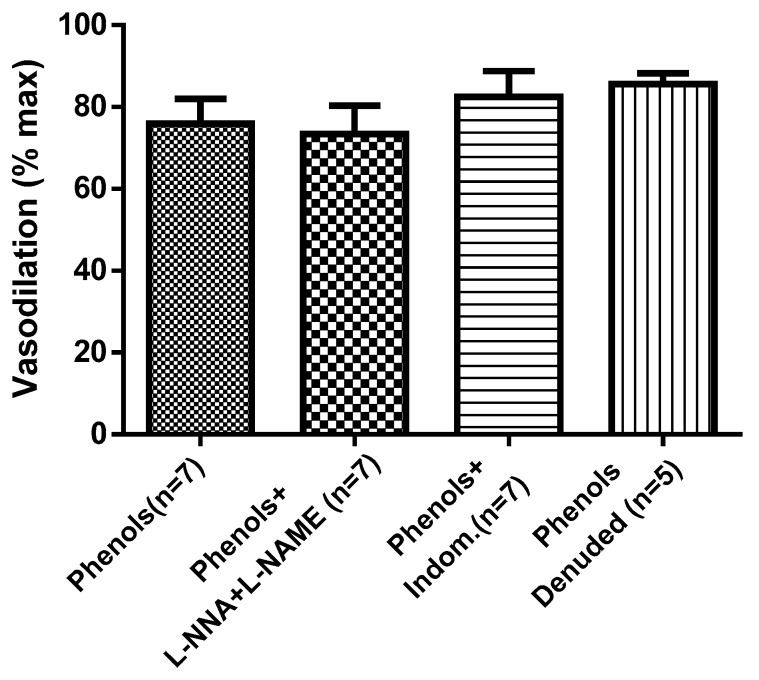
Extra virgin olive oil phenol-induced vasodilation is endothelium-independent. Phenols from crude extra virgin olive oil were tested at 3 × 10^−5^ M on isolated resistance mesenteric arteries in the absence (phenols) and presence of the nitric oxide synthase inhibitors N_ω_-Nitro-l-arginine methyl ester hydrochloride (l-NAME) + N_ω_-Nitro-l-arginine (l-NNA), each at 100 µM, or of the cyclooxygenase inhibitor (Indom, 10 µM). Further, phenols were also tested on the mesenteric artery without the endothelium (denuded). Data are reported as the mean ± SEM, *n* (experimental number).

To find out the molecular mechanism underlying the EVOO-phenol-induced vasodilation, phenols were tested in a single dose (3 × 10^−5^ M) on MA in different conditions. Data in Figure 3 show that removal of the endothelium (denuded artery), inhibition of nitric oxide synthase (NOS) by the inhibitors l-NAME and l-NNA, or inhibition of the cyclooxygenase (COX) by indomethacin did not affect the EVOO-phenol-induced vasodilation, which was similar to that in control vessels.

**Figure 4:**
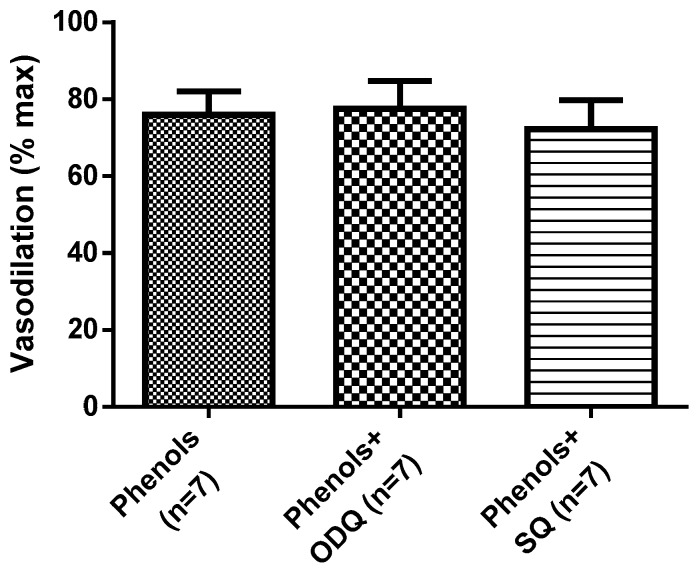
Extra virgin olive oil phenol-induced vasodilation is independent of cyclic nucleotides. Phenols from crude extra virgin olive oil were tested at 3 × 10^−5^ M on isolated resistance mesenteric arteries in the absence (phenols) and presence of the guanilate cyclase inhibitor, 1*H*-[1,2,4]Oxadiazolo[4,3-a]quinoxalin-1-one (ODQ, 10 µM) or the adenilate cyclase inhibitor, 9-(Tetrahydro-2′-furyl)adenine (SQ, 10 µM). Data are reported as mean ± SEM, *n* (experimental number).

Further, EVOO phenols were also tested on MA in the presence of the guanilate cyclase inhibitor, ODQ, or in the presence of the adenilate cyclase inhibitor, SQ. Neither inhibition of cyclic adenosine monophosphate (cAMP) nor cyclic guanosine monophosphate (cGMP) production interfered with the vasodilation induced by EVOO phenols (Figure 4).

**Figure 5:**
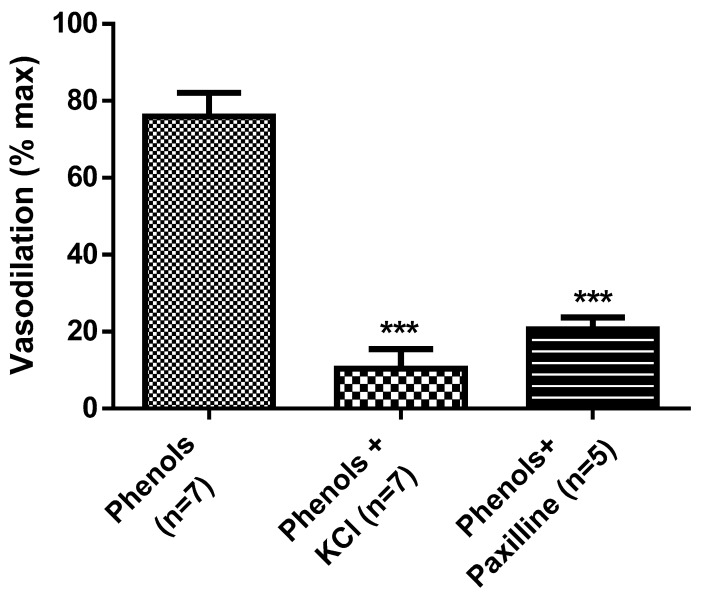
Extra virgin olive oil phenol-induced vasodilation is mediated by hyperpolarization. Phenols from crude extra virgin olive oil were tested at 3 × 10^−5^ M on isolated resistance mesenteric arteries in the absence (phenols) or presence of the BK_Ca2+_ channels inhibitor, paxilline (10 µM, phenols + paxilline). Further, Phenols were tested also on mesenteric arteries depolarized by KCl (40 mM, phenols + KCL). Data are reported as the mean ± SEM, *n* (experimental number). Statistical analysis was performed using Student’s t-test, *** *p* < 0.001.

To determine if EVOO-phenol-induced vasodilation was mediated by hyperpolarization, phenols were tested in MA contracted by KCl, showing that the vasodilation was significant (*p* < 0.001) decreased (Figure 5). Further, the results in Figure 5 show that EVOO-phenol-induced vasodilation of 76% ± 6.1% was significant reduced to 21% ± 2.6% (*p* < 0.001) in the presence of the BK_Ca2+_ channel inhibitor, paxilline.

## 3. Discussion

This study showed a potent vasodilation effect of EVOO phenols on resistance rat MA mediated by activation of BK_Ca2+_ channels in SMCs. The EVOO-phenol-induced vasodilation was endothelium-independent, was not mediated by NO or prostanoids, and did not involve the cyclic nucleotides cAMP or cGMP.

Studies in animals and in humans have shown that a diet rich in EVOO reduces blood pressure [14,15,16] and the hypotensive effect of the EVOO has been attributed to its phenols component [5,7,9,10,14], for which multiple pharmacological effects have also been shown, such as anti-inflammatory [17,18], antioxidant, and radical scavenging activities [19]; antithrombotic effects [20]; and improved endothelial function [21]. The current evidence is strengthened by the fact that intake of phenols, assessed via total phenols excreted (TPE) in urine, was negatively associated with BP levels in a population at high cardiovascular risk [7]. In addition, vasorelaxant properties have been shown in conductive vessels [22,23], due to different and often not yet completely clarified mechanisms of action.

To investigate the potential hypotensive effect, we tested EVOO phenols on small MA, which as a resistance artery their vascular tone determines peripheral vascular resistance, contributing to the regulation of blood pressure and blood flow to organs. The mesenteric vasculature represents about one-third of the total peripheral vascular resistance, and therefore has a consistent influence on the regulation of blood pressure. The authors designed a series of experiments to find out the effect of EVOO phenols on the vascular tone of resistance MA and the possible vascular pathways involved. Our results showed that EVOO phenols dilated the MA in a dose-dependent manner and exerted their maximum action within thirty minutes. Because the ethanol used as the vehicle for the EVOO phenols did not influence vascular tone of the MA, the vasodilation was attributed completely to the phenols. The vasodilation was also maintained when the endothelium was removed from MA, suggesting that EVOO phenols acted on SMCs and induced vasodilation in an endothelium-independent manner. A study on the conductive artery in the rat aortic ring showed similar results [22]. Moreover, it has been reported that minor compounds from olive oil and olive leaves, such as oleuropein, were responsible for acute endothelium-independent vasodilatory effects in isolated *spontaneously hypertensive rat* (SHR) aorta [24]. Indeed, it was observed that sustained intake of an oleuropein-enriched olive leaf extract exerts antihypertensive effects on genetic hypertension by improving vascular function [25]. Further, it has been reported that (3,4-dihydroxyphenylethanol elenolic acid (3,4-DHPEA) and 3,4-dihydroxyphenylethanol elenolic acid dialehyde (3,4-DHPEA-EDA), two of the major phenolic compounds found in virgin olive oil, induced endothelium-independent relaxation at higher concentrations [23], while similar effects were observed for other polyphenols at concentrations higher than 1 µM [26].

In agreement, we have also shown that EVOO-phenol-induced vasodilation in MA was not affected by inhibition of the main endothelial-derived relaxation factors NO and PGI2. In addition, the inhibition of the cyclic nucleotides cGMP and cAMP did not alter the EVOO-phenol-induced vasodilation. Together, these results suggest that EVOO-phenol-induced vasodilation of MA did not occur via the canonical pathways of NO-cGMP vasodilation or PGI2-cAMP vasodilation.

Further, our data clearly demonstrated that EVOO-phenol-induced vasodilation was mediated by hyperpolarization mediated by the BK_Ca2+_ channels, since the vasodilation was abrogated by the very selective BK_Ca2+_ inhibitor paxilline [27]. Several studies using the specific patch clamp technique have shown that BK_Ca2+_ channels are expressed in systemic vascular SMCs, including the mesenteric artery [28,29]. These channels importantly contribute to the steady-state contraction of the SMCs that make up the wall vessels and serve as the primary effectors active in the regulation of vascular tone in resistance arteries [30].

Our results are in agreement with previous studies that have shown the involvement of the BK_Ca2+_ channels in the endothelium-independent vasodilation of the flavonoids quercetin, puerarin, naringenin, dioclein, and luteolin, and for the non-flavonoid phenol resveratrol [31,32,33,34,35,36]. Therefore, activation of BK_Ca2+_ channels seems to be a key mechanism that might account for a good portion of the observed phenol-induced vasodilation.

In this study, we evaluated the effect of the total fraction of phenols in the EVOO. It would be interesting to determine the contributions of the single phenols present in the EVOO. Additionally, another question to address in the future will be to find out if the vasodilation reported in this study results from a synergic effect of the total fraction of EVOO phenols. Further, a future study is required to determine the complete molecular mechanism underlying the actions of EVOO phenols.

In conclusion, for the first time our study demonstrated that EVOO phenols can reduce the vascular tone of resistance arteries and showed the underlying molecular mechanisms by which these compounds could counteract EVOO’s hypotensive properties. Our data support the use of EVOO phenols as a valuable approach for the treatment of hypertension and strengthen clinical evidence recommending the use of EVOO rich in phenols as a possible natural treatment of and preventative approach toward cardiovascular diseases.

## 4. Materials and Methods

### 4.1. Extra Virgin Olive Oil Phenols

#### 4.1.1. Extraction

The phenolic fraction of the EVOO was obtained by solid-phase extraction (SPE) using LiChrolut RP18 cartridges (40–63 mm, 1000 mg/6 mL PP-tubes, Merck, KGaA, Darmstadt, Germany). An SPE cartridge was placed in a vacuum elution apparatus and conditioned by the consecutive addition of 2 × 6 mL of ethanol and 2 × 6 mL of *n*-hexane. EVOO (1 g) was dissolved in 6 mL of *n*-hexane and applied to the column, then the solvent was pulled through, leaving the sample on the solid phase. The sample container was washed prior with 3 × 6 mL of *n*-hexane and then with 3 × 6 mL of ethanol. The ethanolic phase was evaporated in a rotary vaporizer (R-300, Buchi, Uster, Switzerland) at 35 °C, then the dry residue was dissolved with 1 mL of ethanol and filtered through a 0.45-μm pore size nylon filter [28] and stored at −20 °C.

#### 4.1.2. Colorimetric Determination

The ethanolic phase (0.2 mL) was diluted with water to a total volume of 2.5 mL, followed by the addition of 0.25 mL Folin–Ciocalteu reagent [29]. After 3 min, 0.5 mL of Na_2_CO_3_ solution (35%, *w*/*v*) was added to the reaction mixture, which was then mixed and diluted with water to 5 mL. The spectrophotometric measurement was performed at 725 nm after waiting for 2 h against a blank (reaction mixture) sample using a JASCO V-530 spectrophotometer (Champaign, IL, USA) [29]. A calibration curve was calculated using pure oleuropein (Extrasynthèse, ZI Lyon-Nord, Genay, France) in the concentration range of 1–5 mg/L. Oleuropein was used as the standard to dose the fraction of total phenols isolated from the EVOO, because it is the most abundant phenol in EVOO and in oleuropein derivatives.

#### 4.1.3. Animals

All experiments were conducted in accordance with the European Guidelines for the Care and Use of Laboratory Animals (Directive 2010/63/EU) and were approved by the Italian Institutional Animal Care act (130221767483/AR). Sprague–Dawley rats were housed at the University of Calabria Small Animal Facility under controlled conditions on a 12-h light/dark cycle and provided commercial chow and tap water ad libitum. Experiments were performed on male Sprague–Dawley rats at 12–15 weeks of age. Animals were euthanized with isoflurane, followed by decapitation with a small animal guillotine. The abdominal cavity was then opened and a section of the mesentery 5 cm distal to the pylorus was excised and pinned in a Sylgard-lined Petri dish containing cold (4 °C) 4-(2-Hydroxyethyl)piperazine-1-ethanesulfonicacid, *N*-(2-Hydroxyethyl)piperazine-*N*′-(2-ethanesulfonic acid) (HEPES)-physiological saline solution (HEPES-PSS) at pH = 7.4.

#### 4.1.4. Isolated Vessel Preparation

Third-order MA were dissected free of surrounding adipose and connective tissue, cannulated in the chamber of an arteriograph (Instrumentation and Model Facility, University of Vermont, Burlington, VT, USA), and pressurized using a pressure servo system (Living Systems Instrumentation, St Albans City, VT, USA). The intraluminal diameter was measured using a video dimension analyzer (Living Systems Instrumentation) and recorded on LabView software (National Instruments, Austin, TX, USA).

Some experiments were carried out in MA without the endothelium (denuded artery), which was mechanically removed (for details, see [30]), and the successful elimination of the endothelium was verified by the complete loss of acetylcholine (ACh)-induced relaxation. If no vasodilation occurred within five minutes, the vessel was considered suitable, otherwise it was discharged.

#### 4.1.5. Reactivity Study

All vessels were pressurized at an intraluminal pressure of 50 mmHg (as this approximates in vivo conditions), equilibrated for 45 min in HEPES-PSS at 37 °C, and preconstricted with phenylephrine to produce a 40–60% reduction in lumen diameter [31]. Preconstricted MAs were tested with increasing concentrations (10^−9^–3 × 10^−5^ M) of EVOO phenols, and the resulting changes in diameter were recorded once dilation stabilized at each concentration. At the end of each experiment, vessels were treated with relaxing solution containing a mixture of the L-type Ca^2+^ channel blocker diltiazem (10 µM) and the phosphodiesterase inhibitor papaverine (100 µM) to assure maximal vasodilation.

To investigate the molecular mechanism underlying the EVOO-phenol-induced vasodilation, phenols (3 × 10^−5^ M) were tested in MAs that were pretreated for 20 min with the singular following inhibitors prior to exposure to phenylephrine: (1) indomethacin at 10 µM [32] for cyclooxygenase (COX); (2) N_ω_-Nitro-l-arginine (L-NNA) at 100 µM plus N_ω_-Nitro-l-arginine methyl ester hydrochloride (l-NAME) at 100 µM [33], a combination that is more effective in inhibiting nitric oxide synthase (NOS) than either drug alone [34]; (3) ODQ at 10^−5^ M [35] for guanylate cyclase; (4) SQ at 10^−5^ M [36] for adenylate cyclase; and (5) paxilline (10^−5^ M) for BK_Ca_ channels. The inhibitors reported above did not have any significant effect on the phenylephrine contraction. Further, MAs were preconstricted by 40–60% with a high potassium (40 mM) depolarizing solution prior to exposure to EVOO phenols at 3 × 10^−5^ M.

### 4.2. Drugs and Solutions

The HEPES-PSS contained the following (in mmol/L): sodium chloride 141.8, potassium chloride 4.7, magnesium sulfate 1.7, calcium chloride 2.8, potassium phosphate 1.2, HEPES 10.0, EDTA 0.5, and dextrose 5.0. The solutions were prepared in deionized water and titrated with sodium hydroxide (HEPES-PSS) to a physiologic pH of 7.4. Chemicals were purchased from Sigma-Aldrich (Milan, Italy), Fisher Scientific (Milan, Italy), Cayman Chemical Co. (Hamburg, Germany), unless otherwise specified.

All drugs tested were administered from stock solutions prepared daily, except for EVOO phenol stock solutions, which were frozen in small aliquots.

### 4.3. Statistical Analysis

MA dilation induced by EVOO phenols was expressed as a percent of the maximal diameter, which was determined in the presence of the relaxing HEPES-PSS solution. Data are expressed as means ± SEM, where n is the number of arterial segments studied. The *n* values refer to both the number of vessels and number of animals. A normal distribution for all datasets was assumed and differences in responses between groups were determined with two-way ANOVA for repeated measures analysis or by Student’s t-test, as indicated in figure legends. Differences were considered significant at *p* ≤ 0.05.
